# Sex differences in life history, behavior, and physiology along a slow-fast continuum: a meta-analysis

**DOI:** 10.1007/s00265-018-2534-2

**Published:** 2018-07-17

**Authors:** Maja Tarka, Anja Guenther, Petri T. Niemelä, Shinichi Nakagawa, Daniel W.A. Noble

**Affiliations:** 1 Centre for Biodiversity Dynamics, Department of Biology, Norwegian University of Science and Technology (NTNU), Høgskoleringen 5, 7491 Trondheim, Norway; 2 Molecular Ecology and Evolution Lab, Lund University, Ecology building, SE-223 62 Lund, Sweden; 3 Department of Evolutionary Biology, Bielefeld University, Morgenbreede 45, 33615 Bielefeld, Germany; 4 Department of Animal Behaviour, Bielefeld University, Morgenbreede 45, 33615 Bielefeld, Germany; 5 Department of Biology, Ludwig-Maximilians University of Munich, Planegg-, Martinsried, Germany; 6 Ecology and Evolution Research Centre, School of Biological, Earth and Environmental Sciences, The University of New South Wales, Sydney, 2052 Australia

**Keywords:** Life history, Pace-of-life syndrome, Pace-of-life, Phenotypic variation, Sexual selection, Sexual dimorphism

## Abstract

The pace-of-life syndrome (POLS) hypothesis predicts that behavior and physiology covary with life history. Evidence for such covariation is contradictory, possibly because systematic sources of variation (e.g. sex) have been neglected. Sexes often experience different selection pressures leading to sex-specific allocation between reproduction and self-maintenance, facilitating divergence in life-history. Sex-specific differences in means and possibly variances may therefore play a key role in the POLS framework. We investigate whether sexes differ in means and variances along the fast-slow pace-of-life continuum for life history and physiological and behavioral traits. In addition, we test whether social and environmental characteristics such as breeding strategy, mating system, and study environment explain heterogeneity between the sexes. Using meta-analytic methods, we found that populations with a polygynous mating system or for studies conducted on wild populations, males had a faster pace-of-life for developmental life-history traits (e.g., growth rate), behavior, and physiology. In contrast, adult life-history traits (e.g., lifespan) were shifted towards faster pace-of-life in females, deviating from the other trait categories. Phenotypic variances were similar between the sexes across trait categories and were not affected by mating system or study environment. Breeding strategy did not influence sex differences in variances or means. We discuss our results in the light of sex-specific selection that might drive sex-specific differences in pace-of-life and ultimately POLS.

**Electronic supplementary material:**

The online version of this article (10.1007/s00265-018-2534-2) contains supplementary material, which is available to authorized users.

## Introduction

Selection is assumed to favor the integration of life-history and physiology and behavioral traits, mediating the trade-off between current and future reproduction. This so called pace-of-life syndrome (POLS) hypothesis has been proposed as a unifying framework integrating life-history theory with physiology and behavior (Ricklefs and Wikelski 2002; Réale et al. 2010). POLS can be studied at multiple hierarchical levels of biological variation including among-species (Promislow and Harvey 1990), among-populations (e.g. Wikelski et al. 2003), and among-individuals (Réale et al. 2010). Individuals, populations, or species towards the fast end of the pace-of-life (POL) continuum are predicted to have a fast growth rate and invest heavily into early reproduction, resulting in a short lifespan (e.g. Stearns 1983; Promislow and Harvey 1990; Blackburn 1991; Owens and Bennett 1995). In addition, the fast POL strategy is predicted to have weakened immune responses and low physiological as well as behavioral stress responses (Réale et al. 2010). At the same time, individuals following a fast strategy should be aggressive, bold, and explorative, while also exhibiting low levels of parental care (Réale et al. 2010). The opposite trait expressions are expected for the slow end of the POL-continuum. Trait combinations following these predictions have been found between species (Ricklefs 1974; Tieleman et al. 2006; Wiersma et al. 2007; Speakman 2008; Williams et al. 2010; Jimenez et al. 2014) and even among-individuals within populations (e.g. Boon et al. 2007; Dammhahn 2012; Niemelä et al. 2013; Montiglio et al. 2014; Jacques-Hamilton et al. 2017). Nonetheless, these predictions have not been supported across studies (Závorka et al. 2015; Kim and Velando 2016). One explanation for this discrepancy might be that important systematic sources of variation, such as environment (Salzman et al. 2018, topical collection on Pace-of-life syndromes), ecology (Montiglio et al. 2018, topical collection on pace-of-life syndromes), or age- and sex-specific variation (Hämäläinen et al. 2018, Immonen et al. 2018; both in topical collection on Pace-of-life syndromes) have not often been considered.

Due to differences in the potential rate of reproduction, natural and sexual selection are expected to act differently on males and females, driving the evolution of sexual dimorphism (Bateman 1948; Parker 1979; Hedrick and Temeles 1989; Stearns 1992; Andersson 1994; Roff 2002; Fairbairn et al. 2007). Indeed, life-history traits, such as size at birth, growth and maturation rate, reproductive lifespan, and mortality rate commonly differ between the sexes across the animal kingdom (Clutton-Brock and Vincent 1991; Promislow 2003; Fairbairn et al. 2007; Bonduriansky et al. 2008; Maklakov and Lummaa 2013; Adler and Bonduriansky 2014). Assuming that physiological and behavioral traits are aligned with life-history traits on a general POL-continuum, we might predict sex-specific differences in the POL (i.e., sex-specific differences in trait means across all trait categories). Sex differences in POL may be an important driver generating individual-level variation in POLS (i.e., sex-specific trait correlations within populations; e.g., Hämäläinen et al. 2018, Immonen et al. 2018; both in topical collection on pace-of-life syndromes). Indeed, males and females of many species differ in their physiology (e.g., immunology, (Lee 2006; Restif and Amos 2010), metabolism (Nagy 1987; Blaxter 1989; Rønning et al. 2016), hormone profiles (Nelson 2011), and behavior (Smith and Blumstein 2008; Schuett et al. 2010), but the directionality of these differences within and across trait categories has not been formally tested. Fundamentally, differences between males and females in average trait expression may stem from sex-specific reproductive tactics caused by anisogamy (e.g., Bateman 1948; Maynard Smith 1958, 1982; Trivers 1972; Lehtonen et al. 2016; but see Kokko and Jennions 2008). Assuming anisogamy as a naïve null hypothesis would predict that males should be the overall faster sex in life-history, physiology, and behavioral traits. Importantly, however, sex-specific natural selection, as well as sexual selection, may act differently on trait expression and trait associations between the sexes (Arnqvist and Rowe 2005) and could therefore override the faster male POL predicted by anisogamy.

Environmental and social characteristics can generate or alter sex-specific selection that might enhance, mitigate, or even reverse differences dictated by anisogamy in the POL between the sexes (Hämäläinen et al. 2018, topical collection on Pace-of-life syndromes). Breeding biology, such as different mating systems and breeding strategies (semelparity and iteroparity), can affect the intensity of sexual selection (Andersson 1994; Bonnet 2011; Kralj-Fišer et al. 2011; Fisher et al. 2013; Janicke et al. 2016), and thus, the existence of sex-specific POL and POLS. For example, we might expect fast males in polygynous systems, fast females in polyandrous systems, and little differences between the sexes in monogamous systems since the intensity of the sex-specific selection varies across these different mating systems. The environment in which the organisms are studied (i.e., natural habitat or laboratory) can have an effect on selection, e.g., selection can be relaxed or modified in laboratory conditions (Frankham et al. 1986; Pelletier et al. 2009). Such effects may be sex-specific since (i) estimates of life-history are expected to be highly sensitive to environmental variation (absent in laboratory environments) (e.g., Kawasaki et al. 2008), and (ii) extrinsic mortality and mate competition may be lower in laboratory environments (Bonduriansky et al. 2008), potentially leading to differences in the expression of sex-specific POL and POLS between studies conducted in laboratory and wild environment.

Another source of systematic variation that might have led to contradictory evidence for POLS could be consistent differences in sex-specific variances across trait categories. Hence, in addition to mean differences, these may be important for interpreting sex-specific POL and POLS. Differences in phenotypic variance might indicate past episodes of sex-specific selection or be the result of stronger condition dependence (e.g., in the sex with a faster growth rate or larger body size; Rowe and Houle 1996; Råberg et al. 2005). Larger variances in one sex may also imply greater opportunities for sexual and natural selection which can facilitate the evolution of sex-specific POLS (Hämäläinen et al. 2018, topical collection on Pace-of-life syndromes). It has already been well established throughout the animal kingdom that males on average exhibit a higher variance in reproductive success (Janicke et al. 2016); however, much less is known in other traits. Some results indicate that males exhibit a higher phenotypic variance in morphological and behavioral traits (Wyman and Rowe 2014); however, a systematic review across POLS trait categories has not yet been conducted.

Here, we test whether the sexes generally differ in means and variances of traits that are predicted to fall along a fast-slow POL-continuum using meta-analytic methods. Given that data on sex-specific covariance structures across traits, a requirement for testing sex-specific POLS, is very scarce, we decided to focus on testing for sex-differences in trait categories, which are hypothesized to be part of POLS. Specifically, we tested the following questions: (1) Do males generally show a faster POL in adult life-history (e.g., lifespan), developmental life-history (e.g., developmental time or growth rate), physiological and behavioral traits, as predicted by anisogamy? (2) Do effects depend on the breeding strategy, mating system, or the study environment, as predicted by the literature? (3) Do the sexes generally, or for specific trait categories, differ in the amount of phenotypic variance?

## Methods

### Data collection

In our core search, we collected sex and population-specific phenotypic means and variances of POLS-related traits from published papers citing one or both of the seminal POLS papers by (Ricklefs and Wikelski 2002; Réale et al. 2010) until October 2016. To increase the sample size, we combined these studies with two existing databases collected to explicitly test for sex differences in homologous traits. “Database 1” was constructed to investigate differences in behavioral syndromes between males and females (AG et al. unpublished). This database consists of papers that cited three key reviews in the field of animal personality and behavioral syndrome research (Dall et al. 2004; Sih et al. 2004; Schuett et al. 2010), between the years 2010–2013. “Database 2” was originally constructed to compare male- and female-specific genetic (co)variance–matrices (MT et al. unpublished). Here, four main sources were used: papers cited in Poissant et al. (2009), papers cited in Wyman and Rowe (2014), and searches in Web of Science™ Core Collection and Biosis Previews® on the Web of Science platform. For these searches, the following search topics were used: “genetic correlation*” AND *sex*, refined by science categories: ecology OR genetics heredity OR evolutionary biology (for Web of Science™ Core Collection) and genetics OR evolution and adaptation (for Biosis Previews®), and restricting the search to papers published in 2008–2015.

We collected traits falling into three main POLS-trait categories: life-history and behavioral and physiological traits (Réale et al. 2010). Life-history traits were further divided into (1) developmental life-history traits and (2) adult life-history traits, because their importance for POLS might differ. Developmental life-history traits are life-history traits which are measured in immature, not yet reproducing individuals (growth rates and developmental times), while traits categorized as adult life-history are measured in sexually mature individuals (e.g., lifespan and age at first reproduction). We did not consider morphology, since the POLS hypothesis makes no predictions regarding such traits. A collection of the type of traits under each trait category and their directionality according to the POL-continuum is provided in Table S1.

From the databases, we excluded papers that: (1) were not empirical studies (e.g., reviews without data), (2) only included one sex in the analysis or where sexes were treated differently (e.g., subject to different experimental treatments), (3) involved an organism that could change sex (e.g., hermaphrodites), (4) studied plants or humans, and (5) only presented model outputs or reported summary statistics that were not convertible to mean and standard deviation. Furthermore, traits that were not on ratio scale or traits that were not convertible to raw scale had to be excluded. Principal component scores are composite variables composed of traits that might be associated with the POL-continuum in different directions and hence their interpretation may be difficult so these were also excluded. Database 1 and 2 also had some specific exclusion criteria (see Figs. S2–S3).

After removal of duplicates, we had a total of 1431 papers in the three databases (core database 702 papers, database 1 520 papers, database 2 227 papers). Exclusion according to the above-mentioned criteria left us with 109 papers, containing 423 trait values with mean and variance estimates for both sexes (Figs S1-S3). After further scrutinizing the data, 55 estimates from 19 studies were removed because of missing sample sizes, scale issues, or because the trait could not be placed along the fast-slow POL continuum (e.g., lifetime reproductive success). Furthermore, we excluded 10 additional estimates (e.g., song frequency) that were not related to the POLS hypothesis. Finally, we excluded 43 estimates to avoid pseudoreplication as their levels of dependency (see section below) would have been too complex to include in our models, leaving us with 90 papers and 315 trait estimates in our POL database (Fig. S4).

### Effect size statistics

We calculated two different effect size statistics along with their sampling error variance: (1) the log response ratio (lnRR) (Hedges et al. 1999) and (2) log coefficient of variation ratio (lnCVR) (Nakagawa et al. 2015). The lnRR is a ratio between group means, which is free of the effects of group standard deviations (Osenberg et al. 1997; Hillebrand 2008). The lnCVR is the natural logarithm of the ratio between the coefficients of variation, from two groups, enabling a formal comparison of the variability of these groups. We choose to use lnCVR for comparing differences in variance between males and females because it accounts for corresponding changes in the mean; any change in variance could be explained simply by differences in the mean because of the strong mean–variance relationship we observed (Fig. S5; Nakagawa et al. 2015). In our case, our effect sizes were the ratios of the mean (lnRR) and coefficient of variation (CV) (lnCVR) between males and females. Sample sizes and standard deviations for male and female samples do not affect the calculation of effect sizes but are used to calculate each effect size sampling variance (Hedges et al. 1999; Nakagawa et al. 2015). Importantly, for lnRR, we categorized traits as falling along the fast–slow POL continuum in line with predictions from Réale et al. (2010) (see Table S1). Traits for which the directionality was not immediately clear (e.g., walking activity or scale score) were considered only if the authors of the original study indicated the relevance and directionality of the specific trait for the POLS hypothesis. The meaning of larger values for some traits can imply a slow POL (e.g., increased longevity), whereas others (e.g., growth rate or weight gain) would imply a fast POL. As such, we scrutinized individual traits (see Table S1) and inverted directionality of effect magnitude depending on whether higher values of a trait were considered to fall at the fast or slow end of the POL-continuum. In contrast, directionality for lnCVR is independent from the mean and so it was not necessary to invert effect sizes. For both effect sizes, positive estimates indicate faster female POL (lnRR) or larger female variance (lnCVR), whereas negative effect size estimates indicate faster male POL or larger variance in males.

### Impact of mating system, breeding strategy, study environment on POL

Based on predictions in Hämäläinen et al. (2018, topical collection on pace-of-life syndromes), we selected three moderators for which we have sufficient data available that might affect the strength and direction of sex differences in life history and physiological and behavioral traits. We categorized species into different breeding strategies (iteroparous or semelparous) and mating systems (monogamy, polygyny, or promiscuity—only one of the studies had a polyandrous system and was therefore excluded from all analyses). We used social monogamy as a proxy for monogamy, because the majority of studies do not report the genetic mating system. We also categorized the study environment in which the study was conducted (“lab” for populations that have spent at least five generations in a laboratory environment and “wild” for populations studied in the wild or at most for five generations in the laboratory, see Charmantier and Garant (2005) meta-analysis with identical categorization).

When possible, we took the above information for each specific population (i.e., study), but in cases where this was not possible, we used general species information. To extract the species moderator category when it was not available, we searched Web of Knowledge and Google Scholar by combining the moderator term with the species scientific name (e.g., “mating system” AND “*Parus major*”).

### Meta-analytic modeling

We analyzed lnRR and lnCVR using multi-level meta-analytic (MLMA—“intercept only” model) and multi-level meta-regression (MLMR—“predictor” model). Models were run in *metafor* (Viechtbauer 2010) and *MCMCglmm* (Hadfield 2010) to compare results and because Bayesian approaches provide greater flexibility in generating uncertainty around estimates. All models accounted for effect size sampling error variance along with study, species, and phylogenetic non-independence (Hadfield and Nakagawa 2010; Nakagawa and Santos 2012; Noble et al. 2017). We also estimated a residual variance in our MLMA models by including an observation-level random effect to calculate heterogeneity measures as described by Nakagawa and Santos (2012), as *metafor* does not estimate a residual variance by default.

To account for phylogenetic dependencies, we estimated phylogenetic relationships among species using the Open Tree of Life database (https://tree.opentreeoflife.org/; Hinchcliff et al. 2015), which constructs tree topology by synthesizing existing phylogenetic information, using taxonomic relationships when no phylogenetic data is available. From our tree topology (Fig. S6), we estimated a correlation matrix from branch lengths derived using Grafen’s method (Grafen 1989), which is akin to assuming the Brownian model of evolution. Branch lengths were estimated assuming node heights were raised to the power of 0.5. Importantly, Grafen’s method does not account for evolutionary divergence between taxa; however, it does provide a rough estimate of phylogenetic relationships by accounting for phylogenetic topology.

While *metafor* uses frequentist (i.e., likelihood) approaches to estimate parameters, *MCMCglmm* is Bayesian, and requires one to explicitly define prior probability distributions for parameters in the model. As such, we used inverse-Wishart priors (assuming *V* = 1 and nu = 0.002) on all random effects and uniform priors on fixed effects. MCMC approaches estimate parameters through a process similar to stochastic simulation, where parameters in the model are estimated and updated through an iterative sampling process using Bayes theorem. This requires the model to be run iteratively to generate a posterior probability distribution for each parameter. To accomplish this, we ran *MCMCglmm* models for a total of 500,000 iterations, using a burning period of 13,000 iterations. We thinned the MCMC chain by sampling every 100 iterations and ensured that we had an effective sample size for our posterior distribution of greater than 1000. We ensured that a thinning interval of 100 was sufficient by checking the autocorrelation between sampling lags to ensure that values were less than *r* = 0.10. In all cases, results from *MCMCglmm* closely matched results from *metaphor* (results not shown). We present model estimates along with their 95% confidence/credible intervals (CIs) throughout, interpreting estimates with CI’s excluding zero as statistically significant. Overall effects of interest were then back-transformed to percent increases for each sex where appropriate (Nakagawa and Schielzeth 2016). Below, we describe the specific models used to answer our three questions in detail.

#### Models 1 and 2: testing for overall faster and more variable males

To test for existence of overall faster POL in males (model 1), we estimated intercept-only MLMA model using *lnRR* as our response variable as follows:

$$ {lnRR}_i=u+{s}_{k\left[i\right]}+{a}_{p\left[i\right]}+{sp}_{j\left[i\right]}+{m}_i+{e}_i $$

where *u* is the overall mean lnRR; *s*_*k*[*i*]_ is the study-specific random effect, *k*, applied to effect size, *i*; *a*_*p*[*i*]_ is the phylogenetic random effect for species, *j*, applied to effect size, *i*; *sp*_*j*[*i*]_ is the species-specific effect, *k*, applied to effect size, *i*, and accounts for repeated species in the dataset. In contrast, *e*_*i*_ is the observation-level or residual variance for effect size, *i*, and *m*_*i*_ is the effect-size-specific sampling variance, which is known (i.e., inverse sampling variance for each effect size). In our model, all random effects are assumed to be normally distributed with a mean 0 and variance estimated from the model (i.e., ~N(0, ${\sigma}_l^2\boldsymbol{I}$), where ${\sigma}_l^2$ is any of the estimated variance parameters—species identity, study identity, phylogeny, or residual variance and **I** an identity matrix).

To test whether males have increased variance overall compared to females (model 2), independent of any changes in the mean between males and females, we modeled *lnCVR* as follows:

$$ {lnCVR}_i=u+{s}_{k\left[i\right]}+{a}_{p\left[i\right]}+{sp}_{j\left[i\right]}+{m}_i+{e}_i $$

where *u* is the overall mean *lnCVR* and all other variables are defined the same as above.

Using our MLMA models (model 1 and 2), we calculated total heterogeneity (*I*^*2*^_*tot*_) as the proportion of variance among effect sizes after excluding the total sampling error variance (Higgins and Thompson 2002). We also estimated the proportion of variance explained by study (*I*^*2*^_*stdy*_) and phylogeny (*I*^*2*^_*phy*_—representing a phylogenetic signal) as a function of total variance (excluding sampling variance) (Nakagawa and Santos 2012). We calculated heterogeneity estimates from *metafor* (although estimates were similar from *MCMCglmm:* results not shown). To generate confidence intervals on our heterogeneity estimates, we used parametric bootstrapping (*n* simulations = 1500) using variance estimates derived from our MLMA models.

Our data also contained additional sources of non-independence at the within-study level (Nakagawa et al. 2017; Noble et al. 2017). For example, in some studies, different traits were measured on the same individuals (individual-level dependency) at the same time, whereas in other studies, effect sizes were derived from the same individuals measured at different time points (temporal dependency). To test if these additional levels of dependency influenced our results, we modified the identity matrix to a “dependency” matrix, **D**, such that *e*_*i*_ ~N(0, ${\sigma}_e^2\mathbf{D}$), by including correlations at the residual (effect-size) level. Here, ${\sigma}_e^2$ is the residual variance and **D** is a matrix where off-diagonals for correlated effect sizes are assumed to be 0.5 (Noble et al. 2017). We assumed a medium-strength, positive correlation between dependent effect sizes, because the actual correlation was not known. In all cases, including **D** did not impact results from our MLMA models (results not shown), and therefore, we present models without correlation matrices.

#### Models 3–8: effects of mating system, breeding strategy, and study environment

We tested whether sexes differ in the direction of POL between POLS-related trait categories (adult life-history, developmental life-history, physiology and behavior) and whether the moderators affect the strength and direction of sex-specific POL.

We used MLMR models, which accounted for sampling variance, study, and phylogenetic non-independence in *metafor* and *MCMCglmm*. A full model approach was used (included all main effects for each moderator) including (1) trait category (4 levels: adult life-history, developmental life-history, physiology and behavior), (2) the mating system (3 levels: polygyny, promiscuity and monogamy), (3) breeding strategy (2 levels: iteroparity and semelparity), and (4) study environment (2 levels: wild and lab). We only estimated main effects, and not interactions, in our models, given the unbalanced number of trait estimates in many of the categories of the input variables, which would lead to spurious effect sizes. The model was structured as follows:

$$ {ES}_i=u+\boldsymbol{X} \beta +{s}_{k\left[i\right]}+{a}_{p\left[i\right]}+{sp}_{j\left[i\right]}+{m}_i+{e}_i $$

where *ES*_*i*_ is the effect size, either *lnRR* or *lnCVR*; ***Χ*** is the design matrix; and *β* is a vector of estimated coefficients for each of the moderator levels described above. All other variables are described above (see the “Models 1 and 2: testing for overall faster and more variable males” section).

To test the robustness of our results, we again modified the identity matrix to include an assumed correlation (*r* = 0.5—as above) between dependent effect sizes (i.e., replacing **I** with **D**, such that *e*_*i*_ ~N(0, ${\sigma}_e^2\boldsymbol{D}$), see the “Models 1 and 2: testing for overall faster and more variable males” section). However, models ignoring this dependency structure were much better supported (difference between model without and with residual correlation matrix: lnRR-∆AICc = 301.03 for both temporal and individual-level dependency in MLMR models; lnCVR-∆AICc = 20.77 for both temporal and individual-level dependencies). Therefore, we present models assuming effect sizes within studies were independent.

We generated marginal (unconditional) mean estimates, which gives us the mean response for each moderator level, adjusted for any other moderator level in the model. Unconditional means for a given level of a categorical variable (e.g., the level “wild” in the two-level categorical moderator “study environment”) are calculated by averaging (weighted average) the means of this level (i.e., wild) across different combinations of other variables in the model (e.g., each level of trait category: developmental life-history, “adult life-history,” “behavior,” and “physiology”). To accomplish this, and generate credible intervals for these means, we used the posterior distribution from *MCMCglmm*.

It is not yet confirmed whether all subclasses of behavioral or physiological traits are part of the POLS framework. To exclude the possibility that potential effects in trait categories were masked by different or lack of POL-directionality, we also conducted subset analyses [for both lnRR (behavior—model 5, physiology—model 6) and lnCVR (behavior—model 7, physiology—model 8)] using just behavioral and physiological traits. In the first subset analysis, we categorized behavioral traits into measures of “activity,” “aggression,” “boldness,” “exploration,” “parenting,” and “stress-coping” following Réale et al. (2007) (see Table S1). For the second subset analysis, we categorized physiological traits into four categories: immunology, baseline hormone measurement (hereafter “baseline”), and hormones after a stressor was applied (hereafter “stressed”), and “other” for physiology traits not falling into any of the other categories, following Réale et al. (2010).

### Publication bias

We explored evidence for publication bias in lnRR and lnCVR—often resulting from non-significant results not being published (Nakagawa et al. 2017)—by exploring funnel plots of precision (inverse of sampling error variance) versus meta-analytic residuals from intercept-only MLMA. “Meta-analytic residuals” (sensu Nakagawa and Santos 2012) were calculated using *MCMCglmm* (Hadfield 2010) and were conditioned on the random effects accounting for non-independence. Symmetrical funnel plots suggest weak evidence for publication bias; however, funnel plots are often insufficient on their own (Nakagawa et al. 2017). Egger’s regression, in contrast, is a statistical test for funnel plot asymmetry (Egger et al. 1997). It explicitly tests the null hypothesis that the intercept of a regression between effect size residuals (i.e., meta-analytic residuals) standardized by the sampling error against precision (i.e., inverse sampling error variance) is equal to zero (Nakagawa and Santos 2012). A significant intercept is suggestive of publication bias (Egger et al. 1997). While we conducted Egger’s regression with both lnRR and lnCVR, it is important to recognize that there is no clear theoretical reason why lnCVR would be impacted by publication bias, and the utility of methods for assessing publication bias with variance measures has been questioned previously (Senior et al. 2015).

## Results

Overall, we collected *n* = 315 trait mean estimates and standard deviations across 90 studies. Most estimates were derived from physiological traits (*n* = 157), followed by behavioral (*n* = 96), developmental life-history (*n* = 41), and adult life-history traits (*n* = 21). Traits were measured across 69 unique species, with the majority of estimates coming from birds (*n* = 133), followed by insects (*n* = 73), mammals (*n* = 57), fish (*n* = 27), and amphibians and reptiles (*n* = 25). Importantly, most estimates came from systems studied in the wild (lab: *n* = 123; wild: *n* = 192).

### Models 1 and 2: testing for overall faster and more variable males

The overall effect for lnRR (est. = − 0.0498; 95% CI = − 0.1514; 0.0517) and lnCVR (est. = 0.0409; 95% CI = − 0.0054; 0.0873) were both small and not significantly different from zero. Nonetheless, total heterogeneity was high (lnRR: *I*^*2*^_*tot*_ = 0.9990, 95% CI = 0.9988; 0.9991; lnCVR: *I*^*2*^_*tot*_ = 0.9856, 95% CI = 0.9835; 0.9875). We also observed significant study level heterogeneity for both lnRR and lnCVR (lnRR: *I*^*2*^_*stdy*_ = 0.5080, 95% CI = 0.4277; 0.5871; lnCVR: *I*^*2*^_*stdy*_ = 0.1129, 95% CI = 0.0827; 0.1486), even though between-study differences explained little variance for lnCVR. Sub-group analyses for each trait category separately (same MLMA model structure as in Model 1 and 2) also suggested moderate to high between-study variance for developmental life-history and physiological traits, whereas variance among effects in behavioral and life-history traits were primarily driven by within-study effects (Table S2). We only observed a significant, albeit small, phylogenetic signal for lnRR (*I*^*2*^_*phy*_ = 0.0719, 95% CI = 0.0485; 0.0990), whereas we did not observe a phylogenetic signal for lnCVR (*I*^*2*^_*phy*_ = 0).

### Model 3–8: effects of mating system, breeding strategy, and study environment

**Fig. 1:**
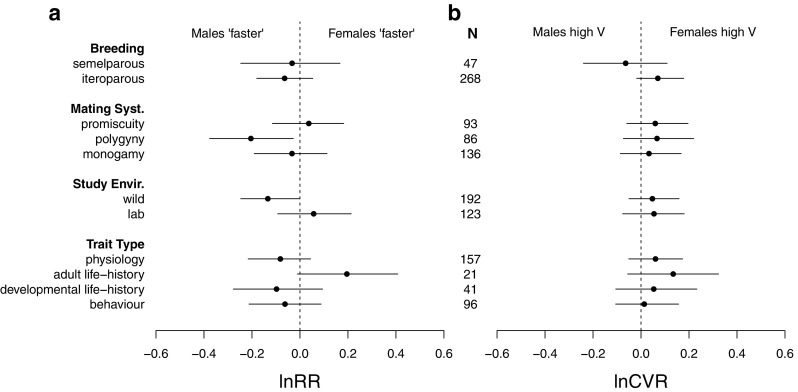
Marginal mean estimates for **a** lnRR and **b** lnCVR. Point estimates and 95% credible intervals are provided. Sample size (*N*) is provided for each level of moderators. Positive values indicate faster POL in females

**Fig. 2:**
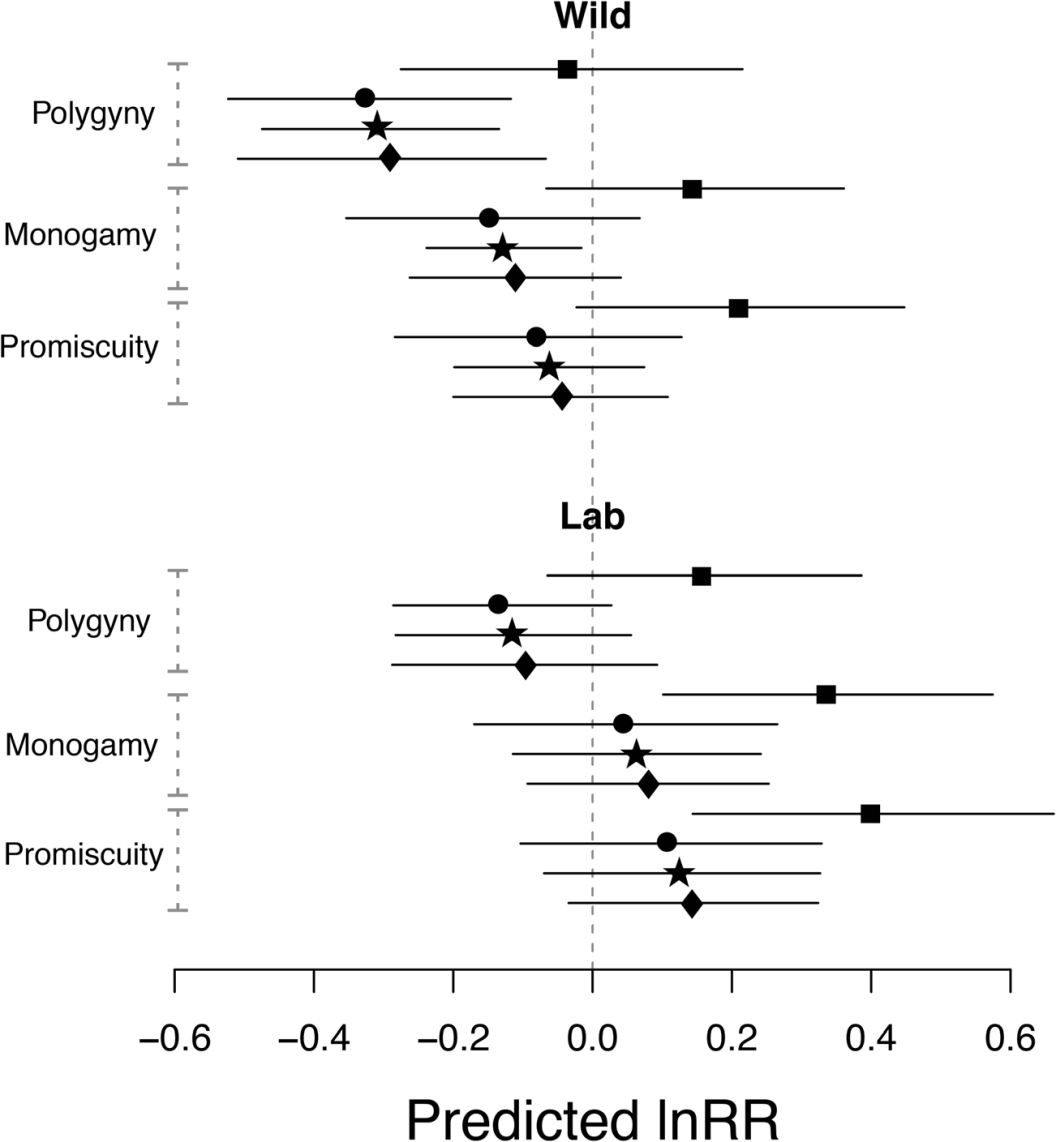
Predicted mean effect size from MLMR model (lnRR) across mating systems and study environment. Means are predicted for an “iteroparous” species. Point symbols correspond to the trait type (square = adult life-history; circle = developmental life-history; star = physiology and diamond = behavior). Point estimates and 95% confidence intervals are provided

Interestingly, males tended to exhibit a faster POL in polygynous mating systems (Fig. 1a—lnRR = − 0.21, 95% CI = − 0.38; − 0.03; see also Table S3 for full model contrast results). This was true for developmental life-history, physiology, and behavior, but not adult life-history, whereas a significant effect did not exist in promiscuous and monogamous mating systems (Fig. 2). Neither mating system nor breeding strategy moderated differences in variance (lnCVR) between the sexes (Fig. 1b, Table S3). Breeding strategy (semelparity/iteroparity) did not moderate the effect sizes (lnRR and lnCVR) (Fig 1a, b).

Studies conducted in the wild had a significant negative lnRR (− 0.135, 95% CI = − 0.272; − 0.02), with males exhibiting a 12% overall faster POL compared to females. This suggests that males in the wild are more likely to exhibit faster POL than females, across averaged trait categories, mating systems, and breeding strategies (Fig. 1a). We did not observe any difference in variance (lnCVR) between wild and laboratory-based studies (Fig. 1b, Table S3).

Accounting for the trait categories and the three moderators, average lnRR showed a small, statistically non-significant, negative effect for behavioral, developmental life-history, and physiological traits (Fig. 1a, for the full model with non-marginalized coefficients; see Table S3), with males exhibiting a 6.22% (behavior) to 9.29% (developmental life-history) faster POL compared to females. The opposite was true of adult life-history traits, which were primarily composed of lifespan (14/21 effect sizes). Here, females exhibited, on average, a 21% faster POL (lnRR = 0.1948, 95% CI = − 0.0096; 0.4164) (Fig. 1a, Table S3). While females appeared to be more variable than males (lnCVR) across traits, these effects were quite small and 95% CI’s overlapped heavily with zero (Fig. 1b, Table S3).

When considering the study environments separately, the trait categories developmental life-history, behavior, and physiology were always clustered, while adult life-history deviates from this pattern (Fig. 2). Furthermore, polygynous mating systems were more likely to have males with traits (at least for developmental life-history, behavior, and physiology) that follow a faster POL, while the effect was weaker (but going in the same direction) in monogamous or promiscuous mating systems. Importantly, this pattern only holds for systems in the wild (Fig. 2). In the lab environment, females exhibit faster life-history across all mating systems.

In the models testing directional heterogeneity in the trait subclasses of behavior and physiology, we generally found no sex-specific differences except for parental care in lnRR (models 5 + 6; Fig. S7A-B, Table 1). Females showed a higher level of parental care, indicative of a slower POL, compared to males (Fig. S7A—lnRR = 0.56, 95% CI = 0.131; 0.960). Trait subclasses showed no effect for lnCVR (Models 7 + 8; Fig. S8A-B, Table 1).

**Table 1:** Coefficients (Est.) and 95% confidence intervals (CI L = lower; CI U = upper) for behavior and physiology subclasses are presented for lnRR and lnCVR. Coefficients come from separate models for the two trait categories (lnRR—model 5 + 6, lnCVR—model 7 + 8). Bolded estimates indicate that confidence intervals do not overlap zero (i.e., are statistically significant). The intercepts refers to the behavioral subclass Activity and the physiological subclass Baseline.

***Behavior***						
	*lnRR*			***lnCVR***		
**Parameter**	**Est.**	**CI L**	**CI U**	**Est.**	**CI L**	**CI U**
*Intercept*	0.200	− 0.095	0.495	0.042	− 0.111	0.196
*Aggression*	− 0.342	− 1.011	0.327	− 0.284	− 0.656	0.089
*Boldness*	− 0.251	− 0.624	0.123	− 0.005	− 0.197	0.188
*Exploration*	− 0.139	− 0.604	0.325	− 0.057	− 0.282	0.168
*Parenting*	− 0.712	− 1.181	− 0.243	− 0.176	− 0.387	0.036
*Stress-coping*	− 0.106	− 0.811	0.598	− 0.012	− 0.388	0.364
***Physiology***						
		*lnRR*			*lnCVR*	
	**Est.**	**CI L**	**CI U**	**Est.**	**CI L**	**CI U**
*Intercept*	− 0.051	− 0.201	0.100	0.089	− 0.056	0.234
*Stressed*	− 0.085	− 0.221	0.051	− 0.077	− 0.280	0.125
*Immune*	0.007	− 0.210	0.225	− 0.141	− 0.368	0.086
*Other*	− 0.092	− 0.277	0.093	− 0.003	− 0.189	0.184

### Publication bias

**Fig. 3:**
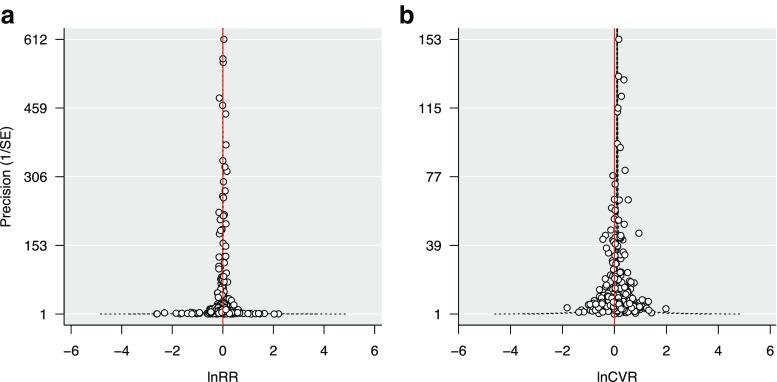
Funnel plots of precision (inverse of sampling standard error) as a function of residuals from meta-analytic models for **a** lnRR and **b** lnCVR. Red vertical line indicates zero effect

We found no evidence for publication bias for lnRR and lnCVR given that meta-analytic residuals showed fairly symmetrical funnel plots (Fig. 3a, b). This was further supported by Egger’s regression with the intercepts not differing significantly from zero (lnRR: df = 313, *t* = − 0.861, *p* = 0.39; lnCVR: df = 313, *t* = − 1.86, *p* = 0.064).

## Discussion

We provide the first quantitative overview testing for differences in the pace-of-life (POL) between the sexes across traits involved in POLS (i.e., adult life-history, developmental life-history, physiology and behavior). Although not exhaustive, our database is based on an unbiased sample of POLS-related studies. Our meta-analysis did not provide general support for overall faster POL in males (i.e., the anisogamy hypothesis), but males exhibit faster POL in polygynous study systems in the wild, at least for developmental life-history and physiological and behavioral traits.

While males often have the potential for a higher reproductive rate than females, our results show that they do not generally exhibit a faster POL in adult and developmental life-history and behavioral and physiological traits. These findings suggest that anisogamy is, as expected, not the only driving force for sex-specific POL and hence potentially also not for POLS. Our results do not suggest that the general predictions about POL differences at the individual level (Réale et al. 2010) can be translated to the between-sex level without considering the social and environmental characteristics of each population or species. Indeed, social and environmental characteristics have also been shown to influence POLS at the among-individual level (Salzman et al. 2018; Montiglio et al. 2018; Royauté et al. 2018; all in topical collection on pace-of-life syndromes).

When looking at patterns in specific POLS-trait categories, we found that behavior, physiology, and developmental life-history cluster together along the slow-fast POL continuum, while adult life-history does not. Faster male development is predicted to increase male resource holding potential, given that sexual selection often acts more strongly on males compared to females (Bateman 1948; Trivers 1972; Janicke et al. 2016; Lehtonen et al. 2016). Here, we find that it does not appear to be a general feature across species in our data. However, males in polygynous mating systems and in studies conducted in the wild have a faster developmental life-history than females (see next section). On the other hand, we also show that females overall had a faster POL in adult life-history (i.e., shorter life). The pace of developmental life-history going against the pace of adult life-history between the sexes contradicts population and species-level predictions from classical life-history theory, which expects short lifespan to be associated with fast growth and early age at maturity (Stearns 1992; Roff 2002; Bonduriansky et al. 2008; Brooks and Garratt 2017). Differences in lifespan between males and females can be both condition- and taxon-dependent and may result from asymmetric inheritance of sex chromosomes, differences in diet and physiology, maternal effects, sexual conflict, and sex-specific selective pressures (Gemmell et al. 2004; Tower and Arbeitman 2009; Maklakov and Lummaa 2013; Adler and Bonduriansky 2014; De Lisle and Rowe 2015; English and Uller 2016), which may explain this unexpected finding. Of course, differences in adult survival may also arise due to early disappearance of one sex from the population. Adult survival estimates in our data comprise a mixture of different taxonomic groups, breeding strategies, and wild and laboratory studies, which should diminish this problem. Nevertheless, the observed differences in adult life-history between the sexes should be interpreted with care, given the small number of effect sizes in our data. Sex-specific differences in adult life-history may affect sex-specific patterns of trait covariance in the context of POLS; however, more empirical research on the magnitude and extent of sex-specific trait covariance patterns across populations and species is needed to investigate this.

Some evidence indicates that males and females frequently differ in the mean expression of behaviors related to POLS (reviewed in Schuett et al. 2010). Sexual selection resulting in different selection pressures between males and females has been argued to explain these differences between the sexes (Andersson 1994; Schuett et al. 2010). However, we did not find any indication that sex-differences in behavior are a general phenomenon across diverse species. The only significant difference between sexes we find is that females generally show more parental behavior compared to males. This is not surprising given that females usually produce far fewer gametes and hence are expected to invest more into post-fertilization care (Trivers 1972). Although differences in parental care may occur for other reasons than the role in life history trade-offs, parental care behavior has been identified as a key driver in linking individual differences in behavior with reproductive success (Mutzel et al. 2013), which suggests a clear link to POLS. Unexpectedly, we also found no indication for sex differences in physiological traits (baseline and challenged HPA-axis related hormones and immune parameters). Sex differences in anatomy and function of HPA-axis regulatory mechanisms are well documented for mammals and stem from sexual dimorphism in the central nervous system affecting physiology and behavior through endocrinology (Rhodes and Rubin 1999). Individual differences in endocrinological processes and traits that are strongly affected by these, e.g. growth rate, have been suggested, and meta-analytically shown, to be the underlying driving forces for consistent individual differences in behavior (Stamps 2007; Biro and Stamps 2008; for meta-analytical approach Niemelä and Dingemanse 2018). In addition, these processes have also been proposed to underlie sex differences in POLS (Immonen et al. 2018). Our results, however, suggest that sex-differences in the HPA-axis related hormone concentrations cannot be generalized across taxa. Sex differences in immunity are also often reported for mammals and birds (Demas and Nelson, 2012; Roved et al. 2016). Although sexual selection affecting the trade-off between reproduction and self-maintenance is assumed to influence male immune responses negatively, we found no sex-differences.

The sex-specific POL predicted by anisogamy can be altered or even overridden by contrasting selection created by, for example, different mating systems or breeding strategies (Hämäläinen et al. 2018, topical collection on pace-of-life syndromes). Indeed, we found that males of polygynous mating systems showed a faster POL than females, as predicted, presumably because sexual and/or natural selection is stronger on males in polygynous species (Greenwood 1980; Wingfield et al. 1990; Clutton-Brock and Isvaran 2007; Brooks and Garratt 2017). On the other hand, social monogamy, often entailing biparental care, is thought to reduce the level of antagonistic selection and conflict between the sexes, leading to more similar life-history optima (Klug et al. 2013). Monogamy is therefore predicted to result in reduced dimorphism of POLS traits, such as lifespan or development (Promislow 1992; Liker and Szekely 2005)—a pattern supported by our results. Also, no sex differences were found in promiscuous mating systems, although we would have expected a more pronounced sexual dimorphism driven by a strong sexual conflict (Hämäläinen et al. 2018, topical collection on pace-of-life syndromes). The sexual conflict should be particularly strong in promiscuous mating systems where males harass females into matings, in opposite to systems where females mate voluntarily, in which case we would expect less dimorphic POLS traits between the sexes. If our database contains a mixture of these two types of promiscuous mating systems, this could explain the lack of sex differences in this group. We therefore advice future studies to specifically look into these differences. Another potential confounding factor might be the presence or absence of parental care. In promiscuous insect systems, where parental care is most often absent, sexual conflict can be mitigated, leading to more monomorphic POL strategies, while in promiscuous mammals and birds, where parental care is female biased, sex differences should be attenuated (Schlicht and Kempenaers 2013; Clutton-Brock 2016). The number of possible breeding attempts per life time combined with promiscuity is also expected to mitigate POL differences between sexes (Hämäläinen et al. 2018, topical collection on Pace-of-life syndromes). In our data, breeding strategy, averaged across all mating systems, did not affect POL in the sexes.

Our results did show a general effect of study environment, with males in the wild having a faster POL. This may be explained by the “robust-male hypothesis” (Bonduriansky et al. 2008), if males in the wild experience higher mortality rates. This hypothesis states that the sex experiencing higher extrinsic mortality in the wild will experience higher condition-dependent selection when brought into benign laboratory conditions, potentially reversing the POL between males and females (Hämäläinen et al. 2014). In artificial environments, trait means might change in an unpredictable way (e.g., individual level behaviors: Niemelä and Dingemanse 2014), because traits measured under laboratory conditions are not under similar constraints or selection, potentially leading to the expression of genetic and phenotypic variation that would have been selected against in the wild (Ghalambor et al. 2007; Schlichting 2008).

We found little evidence for sex-specific effects in phenotypic variances. We could have predicted overall higher variances in males resulting from a number of mechanisms. First, males often are under stronger sexual selection than females, causing a higher variance in reproductive success (Janicke et al. 2016), and potentially therefore also in traits related to reproductive success. Second, males inherit the mitochondrial genome from their mothers, which may contain detrimental alleles that increase variance in male traits (i.e., “Mother’s curse”), particularly in traits related to metabolism (Gemmell et al. 2004; Innocenti et al. 2011). Lastly, stressful environments have been suggested to have important consequences on trait variance (O’Dea et al. 2016), and the sex with higher condition dependence should be more affected by stressful conditions. Males in systems with high male competition (e.g., polygyny) often experience more stressful environments than females and should therefore have a higher variance (e.g., Rowe and Houle 1996). Despite these predictions, we did not find any variance differences between the sexes across any of the trait categories, mating systems, or study environments. If anything, the variances seem to be slightly higher in females. One possible explanation for the lack of a clear pattern in variances could be the mixture of different sex-chromosome systems represented in our data. The sex-chromosome hypothesis states that in species with dosage compensation, the heterogametic sex should show a larger trait variance compared to the homogametic sex (James 1973; Reinhold and Engqvist 2013).

In conclusion, our meta-analysis did not find that males have an overall faster POL, although they were overall faster in polygynous mating systems and in studies conducted in the wild (i.e., more natural environments). This indicates that future studies should take into account the mating system and study environment when trying to understand sex-specific patterns in POL and POLS. Another promising avenue for future research is also to look at how such social and environmental factors interact and affect sex-specific POL and POLS. Surprisingly, we found that adult life-history deviates from all the other trait categories (behavior, physiology and developmental life-history), which all cluster together along the fast-slow POL continuum. In the POLS hypothesis, life history traits play a key role because the framework aims to predict how individuals and sexes mediate the trade-off between current and future reproduction (Dammhahn et al. 2018, topical collection on Pace-of-life syndromes). Thus, life history traits must always be included in studies investigating POLS. Our data clearly show that future studies need to acknowledge that the conclusion about POLS might differ depending on whether the chosen life-history trait represents developmental or adult life-history traits. The lack of differentiation between sexes in the POLS literature, so far, may have been caused by a general focus on females in the classical life-history literature. Our results encourage a more thorough investigation into sex-specific POL, how such differences translate into different pace-of-life syndromes, and the implications of these trait covariances for population ecology and evolution.

## Electronic supplementary material

ESM 1 (DOCX 1136 kb)
