# Supplementary material for: Sex differences in life history, behavior, and physiology along a slow-fast continuum: a meta-analysis
Source: Behav Ecol Sociobiol. 2018 Jul 17;72(8):132. doi: 10.1007/s00265-018-2534-2 (PMC6060830; doi:10.1007/s00265-018-2534-2)
Supplement: Supplementary file 1 — (DOCX 1136 kb) [file 265_2018_2534_MOESM1_ESM.docx]

**Supplementary material:**

**Sex differences in life-history, behavior and physiology along a** **slow-fast continuum: a meta-analysis**

**Journal: Behavioral Ecology and Sociobiology**

Maja Tarka^1,2^*, Anja Guenther^3,4^*, Petri T. Niemelä^5^, Shinichi Nakagawa^6^ and Daniel W.A. Noble^6^

**^1^ -** Centre for Biodiversity Dynamics, Department of Biology, Norwegian University of Science and Technology (NTNU), Høgskoleringen 5, 7491 Trondheim, Norway

**^2^ -** Molecular Ecology and Evolution Lab, Lund University, Ecology building, SE-223 62 Lund, Sweden

**^3^** - Department of Evolutionary Biology, Bielefeld University, Morgenbreede 45, 33615 Bielefeld, Germany

**^4^**- Department of Animal Behaviour, Bielefeld University, Morgenbreede 45, 33615 Bielefeld, Germany

**^5^-** Department of Biology, Ludwig-Maximilians University of Munich, Planegg-Martinsried, Germany

**^6^ -** Ecology and Evolution Research Centre, School of Biological, Earth and Environmental Sciences, The University of New South Wales, Sydney 2052, Australia

* Both authors contributed equally to this work.

Corresponding author: Maja Tarka, phone: +46 739 498613, email: maja.tarka@biol.lu.se


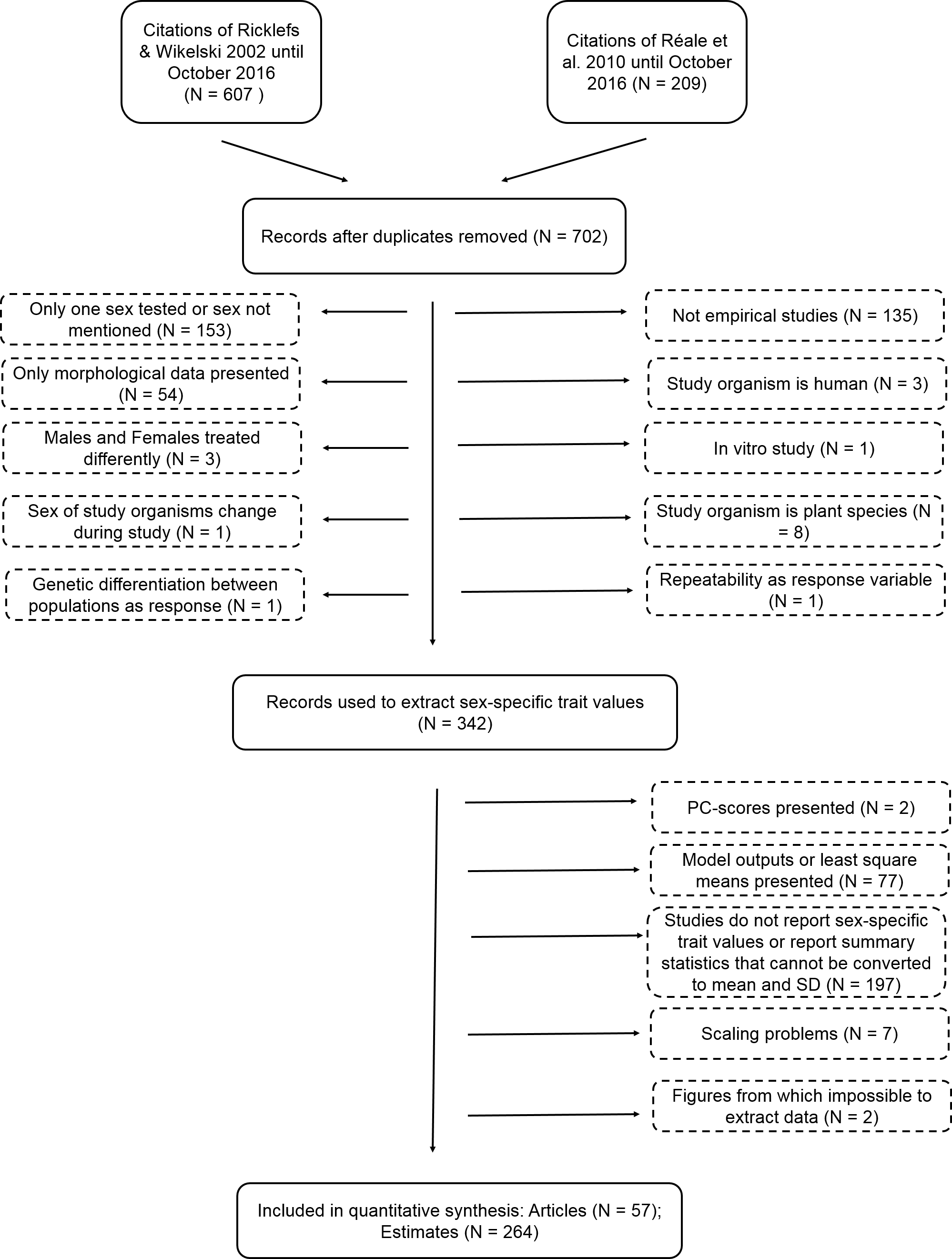


**Fig. S1** PRISMA diagram over study and trait selection process for studies citing the seminal POLS publications Ricklefs and Wikelski (2002) and/or Réale et al. (2010) for the core database


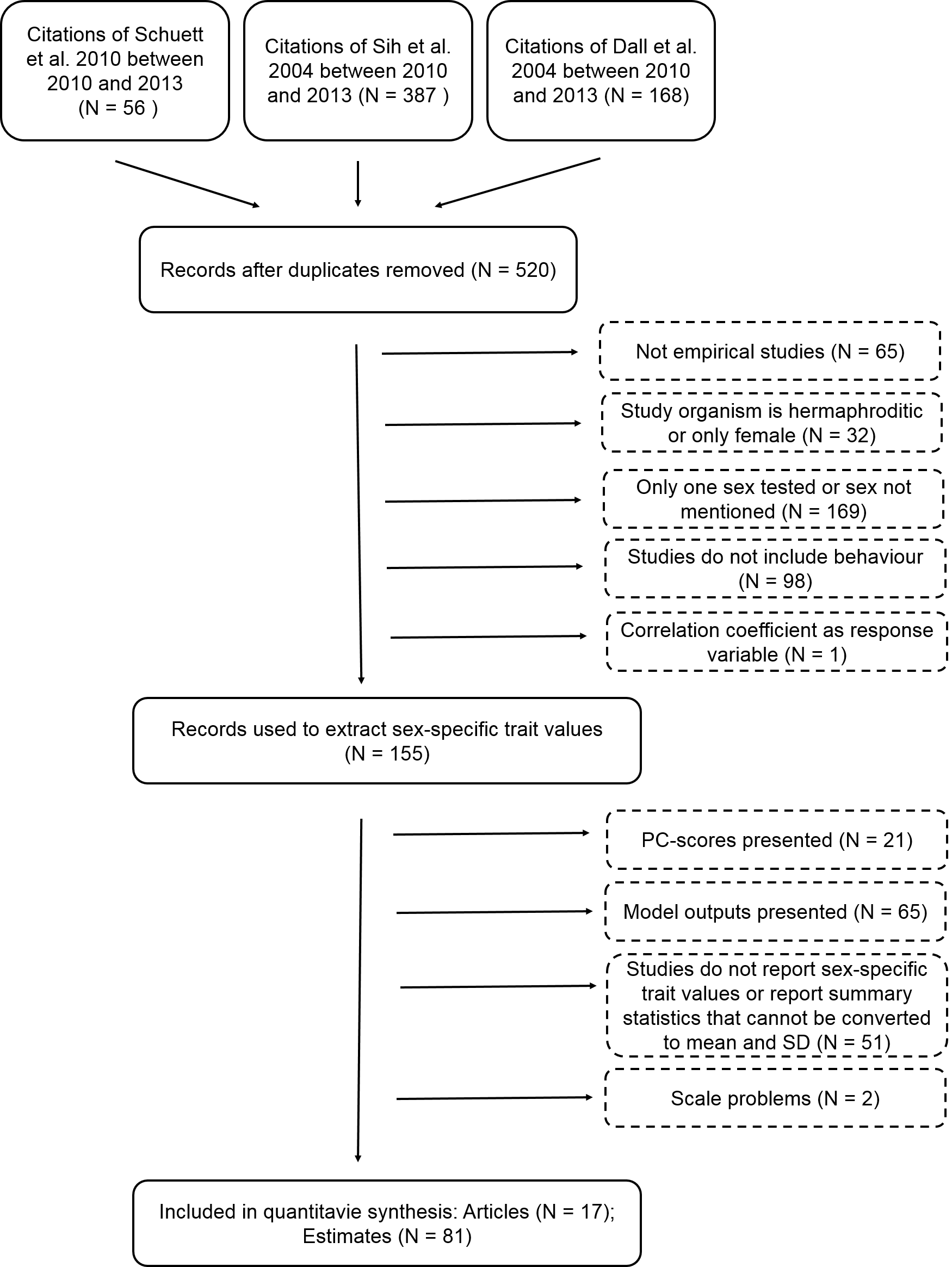


**Fig. S2** PRISMA diagram over study and trait selection process for Database 1


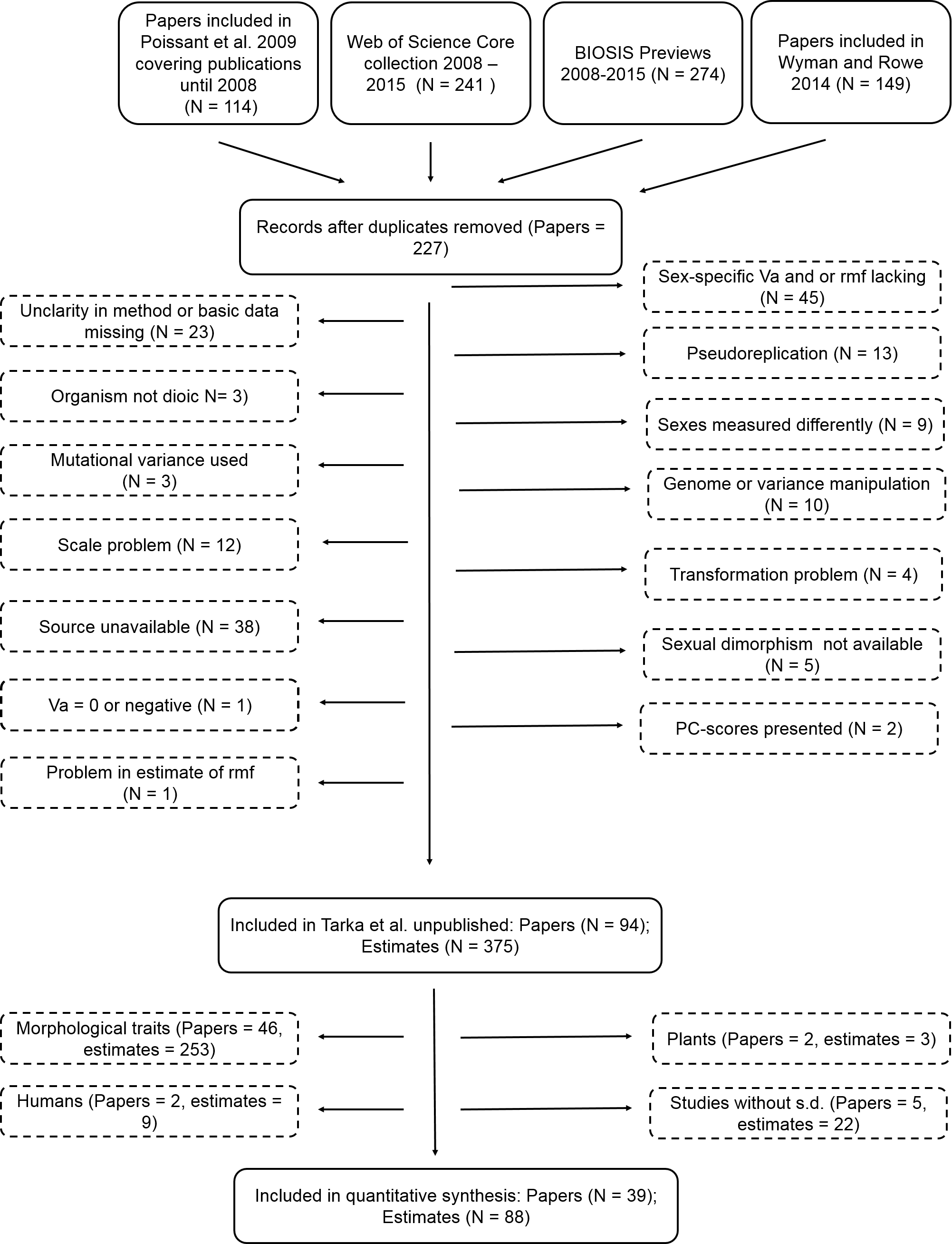


**Fig. S3** PRISMA diagram over study and trait selection process for Database 2


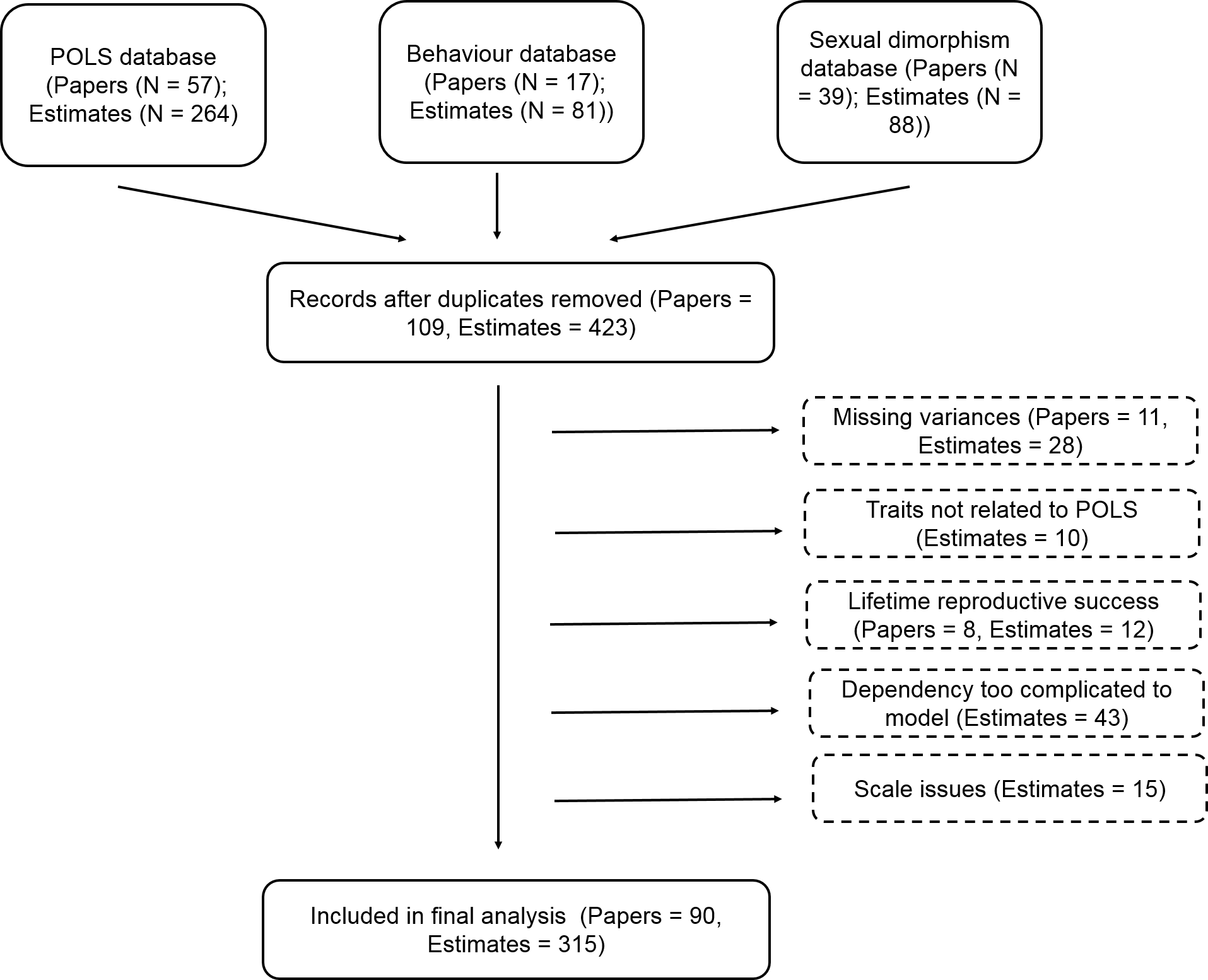


**Fig. S4** PRISMA diagram over merging of the three databases and further pruning to obtain final data used in analyses

**Fig. S5** Mean-variance relationship in males (blue circles) and females (red circles)

**Fig. S6** Phylogenetic tree of taxa included in the meta–analysis

**Fig. S7** Predicted means for behavioral subclass categories from MLMR models. A) lnRR and B) lnCVR Point estimates and 95% confidence intervals are provided. Sample size (N) is provided for each level of moderator. Positive values indicate faster POL (lnRR) or higher variance (lnCVR) in females

**Fig. S8** Predicted means for physiology subclass categories from MLMR models. A) lnRR and B) lnCVR Point estimates and 95% confidence intervals are provided. Sample size (N) is provided for each level of moderator. Positive values indicate faster POL (lnRR) or higher variance (lnCVR) in females

**Table S1** List of traits included in each trait category with POL directionality. a) behavioral and physiological traits with trait classes, b) adult life-history and developmental life-history traits

| **Behavioral traits** | | | **Physiological traits** | | |
| --- | --- | --- | --- | --- | --- |
| Trait | Subclass | Direction | Trait | Subclass | Direction |
| Activity level, attractive novel object | activity | high values = fast | Baseline corticosterone | baseline | high values = slow |
| Activity level, barrier threat | activity | high values = fast | Baseline cortisol | baseline | high values = slow |
| Activity level, familiar flock | activity | high values = fast | Baseline prolactin | baseline | high values = slow |
| Activity level, unattractive novel object | activity | high values = fast | Corticosterone | baseline | high values = slow |
| Activity level, unfamiliar flock | activity | high values = fast | Agglutination | immune | high values = slow |
| Activity | activity | high values = fast | Antibody responsiveness | immune | high values = slow |
| Climbing | activity | high values = fast | LPS antibody titre | immune | high values = slow |
| Flight activity | activity | high values = fast | Lymphocyte count | immune | high values = slow |
| Load score (activity) | activity | high values = fast | Lysis | immune | high values = slow |
| Locomotor activity | activity | high values = fast | Oxidative burst | immune | high values = slow |
| Net distance travelled | activity | high values = fast | Percentage of bacteria killed | immune | high values = slow |
| Open field activity | activity | high values = fast | PHA antibody titre | immune | high values = slow |
| Scale score (activity) | activity | high values = fast | PHA swelling response | immune | high values = slow |
| Total distance travelled | activity | high values = fast | Phagocyte count | immune | high values = slow |
| Turning around | activity | high values = fast | PHA-induced wing-web thickness | immune | high values = slow |
| Walking backwards | activity | high values = fast | Primary (anti-BGG) antibody response | immune | high values = slow |
| Aggression | aggression | high values = fast | Summer fecal egg count | immune | high values = fast |
| No. Of aggression | aggression | high values = fast | Total imunoglobulin G level (IgG) | immune | high values = slow |
| Number of agonistic interactions | aggression | high values = fast | ADFI average daily feed intake | other | high values = fast |
| Boldness | boldness | high values = slow | ADG average daily gain | other | high values = fast |
| Duration of tonic immobility | boldness | high values = slow | Androgen levels | other | high values = fast |
| Emergence time | boldness | high values = slow | Average daily food intake Env 3 | other | high values = fast |
| Grooming duration | boldness | high values = fast | BMR | other | high values = fast |
| Latency to autotomize limb | boldness | high values = fast | Early molt score | other | high values = fast |
| Latency to cross barrier | boldness | high values = slow | Endurance: time to exhaustion | other | high values = slow |
| Latency to eat unknown food item | boldness | high values = slow | FCR feed conversion ratio | other | high values = slow |
| Latency to emerge | boldness | high values = slow | Food conversion ratio | other | high values = slow |
| Latency to resume eating | boldness | high values = slow | Glutathione peroxidaze (GPx) activity | other | high values = slow |
| Latency to touch object | boldness | high values = slow | Heat resistance | other | high values = slow |
| Lines crossed in open field | boldness | high values = fast | Heat shock resistance | other | high values = slow |
| Number of strokes | boldness | high values = fast | Hemocyte count | other | high values = slow |
| Shoaling with opposite sex (first move) | boldness | high values = slow | Oxidative status (OS) | other | high values = fast |
| Shoaling with opposite sex (time to surface) | boldness | high values = slow | Phenoloxidase activity | other | high values = slow |
| Shoaling with same sex (first move) | boldness | high values = slow | Plasma antioxidant capacity (OXY) | other | high values = slow |
| Shoaling with same sex (time to surface) | boldness | high values = slow | Plasma hydroperoxides (ROMs) | other | high values = fast |
| Time spend active | boldness | high values = fast | Resting metabolic rate | other | high values = fast |
| Time spend in the water surface | boldness | high values = fast | Serum bone-specific alkaline phosphatase | other | high values = fast |
| Waling activity | boldness | high values = fast | Spleen SOD | other | high values = slow |
| **Table S1 continued** |  |  |  |  |  |
| Dispersal distance | dispersal | high values = fast | Spleen tGSH | other | high values = slow |
| Inter-annual nest distance | dispersal | high values = fast | Starvation resistance | other | high values = slow |
| Exploration | exploration | high values = fast | superoxide dismutate (SOD) activity | other | high values = slow |
| Feeder exploration | exploration | high values = fast | Testosterone | other | high values = fast |
| Time to approach conspecifics | exploration | high values = slow | Time to reach BMR | other | high values = slow |
| NO score | exploration | high values = fast | 30-min corticosterone | stressed | high values = slow |
| No. of feeders visited in NE | exploration | high values = fast | Corticosterone stress response | stressed | high values = slow |
| No. of fin spreads | exploration | high values = fast | Prolactin stress | stressed | high values = slow |
| No. of fin spreads NE | exploration | high values = fast | Stress recovery 30min prolactin | stressed | high values = slow |
| No. of stopps during exploration | exploration | high values = slow | Stressed cortisol | stressed | high values = slow |
| No. of touched objects | exploration | high values = fast | Total integrated adrenocortical response | stressed | high values = slow |
| Time spent in flower pot | exploration | high values = slow |  |  |  |
| Time spent in hot zone NO | exploration | high values = fast |  |  |  |
| Time spent swimming in NE | exploration | high values = fast |  |  |  |
| Provisioning rate | parenting | high values = slow |  |  |  |
| Direct parental care | parenting | high values = slow |  |  |  |
| Indirect parental care | parenting | high values = slow |  |  |  |
| Nestling provisioning rate | parenting | high values = slow |  |  |  |
| Prey biomass delivered | parenting | high values = slow |  |  |  |
| Provisioning behaviour | parenting | high values = slow |  |  |  |
| Time spent at nest | parenting | high values = slow |  |  |  |
| Fearlessness | stress-coping | high values = fast |  |  |  |
| TI duration | stress-coping | high values = fast |  |  |  |
| Vocal score | stress-coping | high values = slow |  |  |  |

| **Developmental life-history traits** | | **Adult life-history traits** | |
| --- | --- | --- | --- |
| Trait | Direction | Trait | Direction |
| Age (weeks) of peak tarsus length growth rate | high values = slow | Age at first reproduction | high values = slow |
| Average daily gain | high values = fast | Life span | high values = slow |
| Body weight gain from week 3 to 6 | high values = fast | Mating frequency | high values = fast |
| Daily gain on test | high values = fast |  |  |
| Daily weight gain | high values = fast |  |  |
| Days from 20 to 100 kg | high values = slow |  |  |
| Development time | high values = slow |  |  |
| Maturation | high values = slow |  |  |
| Post-weaning weight gain Simmental | high values = fast |  |  |
| Relative growth | high values = fast |  |  |
| Steepest slope of body mass growth | high values = fast |  |  |
| Steepest slope of tarsus length growth | high values = fast |  |  |
| Weight gain from birth to weaning | high values = fast |  |  |

**Table S2** Within-trait heterogeneity estimates, *I^2^* (Est.) and 95% confidence intervals (CI L = lower; CI U = upper), for lnRR and lnCVR

|  | **lnRR** | | | **lnCVR** | | |
| --- | --- | --- | --- | --- | --- | --- |
|  | *Est.* | *CI L* | *CI U* | *Est.* | *CI L* | *CI U* |
| **Physiology (n = 157)** |  |  |  |  |  |  |
| *I^2^_stdy_* | 0.7037 | 0.5919 | 0.7935 | 0.1806 | 0.1154 | 0.2557 |
| *I^2^_tot_* | 0.9981 | 0.9974 | 0.9986 | 0.956 | 0.9474 | 0.9637 |
| **Developmental LH (n = 41)** |  |  |  |  |  |  |
| *I^2^_stdy_* | 0.5284 | 0.3083 | 0.7208 | 0.7534 | 0.5598 | 0.8796 |
| *I^2^_tot_* | 0.9988 | 0.9981 | 0.9992 | 0.9929 | 0.9877 | 0.9961 |
| **Behavior (n = 96)** |  |  |  |  |  |  |
| *I^2^_stdy_* | 0.2689 | 0.1582 | 0.3840 | 0.0463 | 0.0237 | 0.0746 |
| *I^2^_tot_* | 0.9732 | 0.9660 | 0.9790 | 0.9659 | 0.9561 | 0.974 |
| **Adult LH (n = 21)** |  |  |  |  |  |  |
| *I^2^_stdy_* | 0.0000 | 0.0000 | 0.0000 | 0.0574 | 0.0154 | 0.1388 |
| *I^2^_tot_* | 0.9838 | 0.9697 | 0.9912 | 0.9713 | 0.9488 | 0.9839 |

**Table S3** Coefficients (Est.) and 95% confidence intervals (CI L = lower; CI U = upper) for full models (Models 3 & 4). Bolded estimates indicate that confidence intervals do not overlap zero (i.e., are statistically significant), whereas italicized estimates indicate marginal significance (i.e., 0.1 > p > 0.05). Abbreviations are as follows: *Dev* = Developmental life-history; *LH* = Adult life-history; *Phys* = Physiology; *Promis.* = Promiscuity; *Polyg..*= Polygyny and *Mat.Sys=* mating system. The intercept refers to Behavior in iteroparous, monogamous species in lab environment

|  | ***lnRR*** | | | ***lnCVR*** | | |  |
| --- | --- | --- | --- | --- | --- | --- | --- |
| **Parameter** | **Est.** | **CI L** | **CI U** | **Est.** | **CI L** | **CI U** | |
|  |  |  |  |  |  |  | |
| *Intercept* | 0.080 | -0.093 | 0.252 | -0.014 | -0.139 | 0.112 | |
| *Trait Type (Dev)* | -0.032 | -0.237 | 0.173 | 0.048 | -0.122 | 0.217 | |
| *Trait Type (LH)* | **0.258** | **0.035** | **0.482** | 0.129 | -0.058 | 0.317 | |
| *Trait Type (Phys)* | -0.016 | -0.164 | 0.131 | 0.041 | -0.079 | 0.161 | |
| *Wild* | **-0.191** | **-0.345** | **-0.036** | -0.004 | -0.125 | 0.117 | |
| *Mat.Sys (Polyg.)* | *-0.177* | *-0.364* | *0.009* | 0.051 | -0.089 | 0.191 | |
| *Mat.Sys (Promisc.)* | 0.065 | -0.092 | 0.222 | 0.072 | -0.050 | 0.195 | |
| *Breeding (semelparous)* | 0.032 | -0.157 | 0.221 | -0.120 | -0.265 | 0.026 | |
|  |  |  |  |  |  |  | |
